# A Genome Epidemiological Study of SARS-CoV-2 Introduction into Japan

**DOI:** 10.1128/mSphere.00786-20

**Published:** 2020-11-11

**Authors:** Tsuyoshi Sekizuka, Kentaro Itokawa, Masanori Hashino, Tetsuro Kawano-Sugaya, Rina Tanaka, Koji Yatsu, Asami Ohnishi, Keiko Goto, Hiroyuki Tsukagoshi, Hayato Ehara, Kenji Sadamasu, Masakatsu Taira, Shinichiro Shibata, Ryohei Nomoto, Satoshi Hiroi, Miho Toho, Tomoe Shimada, Tamano Matsui, Tomimasa Sunagawa, Hajime Kamiya, Yuichiro Yahata, Takuya Yamagishi, Motoi Suzuki, Takaji Wakita, Makoto Kuroda

**Affiliations:** a Pathogen Genomics Center, National Institute of Infectious Diseases, Tokyo, Japan; b Sapporo City Institute of Public Health, Sapporo, Japan; c Ibaraki Prefectural Institute of Public Health, Ibaraki, Japan; d Gunma Prefectural Institute of Public Health and Environmental Sciences, Gunma, Japan; e Saitama Prefectural Institute of Public Health, Saitama, Japan; f Department of Microbiology, Tokyo Metropolitan Institute of Public Health, Tokyo, Japan; g Division of Virology and Medical Zoology, Chiba Prefectural Institute of Public Health, Chiba, Japan; h Microbiology Department, Nagoya City Public Health Research Institute, Aichi, Japan; i Department of Infectious Diseases, Kobe Institute of Health, Kobe, Hyogo, Japan; j Division of Microbiology, Osaka Institute of Public Health, Osaka, Japan; k Fukui Prefectural Institute of Public Health and Environmental Science, Fukui, Japan; l Infectious Disease Surveillance Center, National Institute of Infectious Diseases, Tokyo, Japan; m National Institute of Infectious Diseases, Tokyo, Japan; University of Michigan—Ann Arbor

**Keywords:** SARS-CoV-2, COVID-19, genome, haplotypes, epidemiology, immigration

## Abstract

After the first case of coronavirus disease 2019 (COVID-19) in Japan on 15 January 2020, multiple nationwide COVID-19 clusters were identified by the end of February. The Japanese government focused on mitigating the emerging COVID-19 clusters by conducting active nationwide epidemiological surveillance. However, an increasing number of cases continued to appear until early April 2020, many with unclear infection routes and no recent history of travel outside Japan. We aimed to evaluate the severe acute respiratory syndrome coronavirus 2 (SARS-CoV-2) genome sequences from the COVID-19 cases that appeared until early April 2020 and to characterize their genealogical networks in order to demonstrate possible routes of spread in Japan. Nasopharyngeal specimens were collected from patients, and reverse transcription-quantitative PCR tests for SARS-CoV-2 were performed. Positive RNA samples were subjected to whole-genome sequencing, and a haplotype network analysis was performed. Some of the primary clusters identified during January and February 2020 in Japan descended directly from the Wuhan-Hu-1-related isolates from China and other distinct clusters. Clusters were almost contained until mid-March; the haplotype network analysis demonstrated that the COVID-19 cases from late March through early April may have created an additional large cluster related to the outbreak in Europe, leading to additional spread within Japan. In conclusion, genome surveillance has suggested that there were at least two distinct SARS-CoV-2 introductions into Japan from China and other countries.

**IMPORTANCE** This study aimed to evaluate the severe acute respiratory syndrome coronavirus 2 (SARS-CoV-2) genome sequences from COVID-19 cases and to characterize their genealogical networks to demonstrate possible routes of spread in Japan. We found that there were at least two distinct SARS-CoV-2 introductions into Japan, initially from China and subsequently from other countries, including Europe. Our findings can help understand how SARS-CoV-2 entered Japan and contribute to increased knowledge of SARS-CoV-2 in Asia and its association with implemented stay-at-home/shelter-in-place/self-restraint/lockdown measures. This study suggested that it is necessary to formulate a more efficient containment strategy using real-time genome surveillance to support epidemiological field investigations in order to highlight potential infection linkages and mitigate the next wave of COVID-19 in Japan.

## Abstract (precis)

This study aimed to evaluate the severe acute respiratory syndrome coronavirus 2 (SARS-CoV-2) genome sequences from COVID-19 cases and to characterize their genealogical networks to demonstrate possible routes of spread in Japan. We found that there were at least two distinct SARS-CoV-2 introductions into Japan, initially from China and subsequently from other countries, including Europe. Our findings can help understand how SARS-CoV-2 entered Japan and contribute to increased knowledge of SARS-CoV-2 in Asia and its association with implemented stay-at-home/shelter-in-place/self-restraint/lockdown measures. This study suggested that it is necessary to formulate a more efficient containment strategy using real-time genome surveillance to support epidemiological field investigations in order to highlight potential infection linkages and mitigate the next wave of COVID-19 in Japan.

## INTRODUCTION

The initial coronavirus disease 2019 (COVID-19) outbreak occurred in Wuhan, China, in late December 2019. It was caused by a new strain of betacoronaviruses known as severe acute respiratory syndrome coronavirus 2 (SARS-CoV-2) (1–3). After the identification of the first patient with COVID-19 in Japan on 15 January 2020, multiple local COVID-19 clusters were identified nationwide by the end of February. The Japanese government focused on identifying and mitigating the emerging COVID-19 clusters before they could spread further. In an effort to contain these clusters and limit the number of new cases, active nationwide epidemiological surveillance of each cluster was conducted in order to identify the close contacts of existing patients with COVID-19. Japan has sustained moderate spread by focusing on COVID-19 outbreak clusters; however, an ever-increasing number of COVID-19 cases appeared until early April, which made it difficult to identify all the infection routes.

Although some of the COVID-19 clusters were successfully contained, the number of cases continued to increase. On 16 April 2020, the Japanese government declared a nationwide state of emergency in view of the worsening spread. To support ongoing epidemiological surveillance, we collaborated with local public health institutes in Japan (see Table S1 in the supplemental material) and conducted whole-genome sequencing of SARS-CoV-2. Our goal was to apply genomic epidemiology to predict potential routes of infection within or between clusters. Thus far, multiple studies have been conducted to demonstrate the spread of COVID-19 using nationwide and global comparative genome surveillance; as of 2 June 2020, there were already 33,483 complete genomes publicly available on the Global Initiative on Sharing All Influenza Data (GISAID) platform (4). Some detailed reports have been conducted to highlight region-specific clusters and intra- and international spread observed in the United States (https://covidgenomics.org/), the United Kingdom (https://www.cogconsortium.uk/), Hungary (5), Australia (6), Denmark (7), Iceland (8), and California (9).

10.1128/mSphere.00786-20.1 TABLE S1 Collaboration with local public health institutes in the COVID-19 Genomic Surveillance Network in Japan. Download Table S1, XLSX file, 0.01 MB. Copyright © 2020 Sekizuka et al. 2020 Sekizuka et al. This content is distributed under the terms of the Creative Commons Attribution 4.0 International license.

In this study, we aimed to evaluate the viral genome sequences from COVID-19 cases that were identified until early April 2020 and characterize their genealogical networks in order to demonstrate possible routes of spread in Japan.

## RESULTS AND DISCUSSION

**FIG 1:**
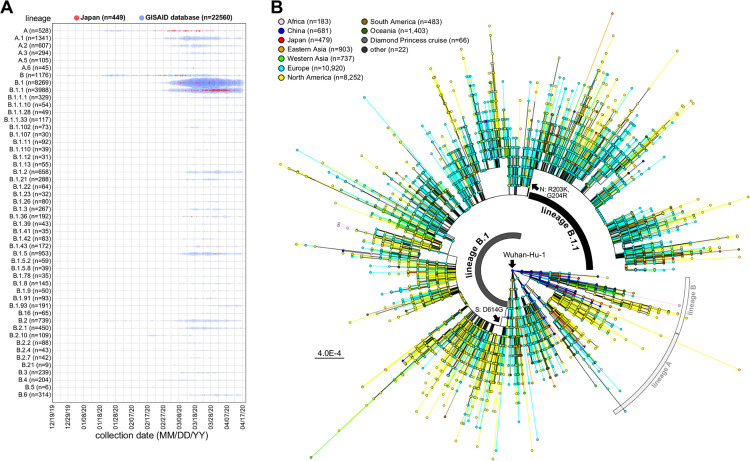
Phylogenetic classification using SARS-CoV-2 genome sequences. (A) Genome lineage classification by the PANGOLIN program (12). One hundred forty-five lineages were detected in 24,129 isolates, including Japanese isolates (*n* = 435), followed by visualization with a bubble chart of the top 50 lineages. The red and blue circles indicate Japanese isolates of this study and available sequences in the GISAID database reported from other countries, respectively. The size of the circle indicates the number of detected isolates. The *x* axis indicates the time scale from 19 December 2019 to 17 April 2020. (B) A maximum likelihood phylogenetic tree was constructed using FastTree-2 and Wuhan-Hu-1 (GISAID accession no. EPI_ISL_402125) as an outgroup reference, which is located at the center point of the radial tree for tree rooting. The geographic and sample information are indicated in the color schemes on the outer slot of the phylogenetic tree.

The nearly full-length genome sequences (≥29 kb; 23,694 entries) of SARS-CoV-2 were retrieved from the GISAID EpiCoV database (collected by 16 April 2020; submitted by 4 October 2020) (4). We determined the full genome sequences using 435 clinical specimens from Japan collected until 6 April 2020 followed by phylogenetic analysis using genome-wide single nucleotide variations (SNVs) to trace potential infection routes (Fig. 1). After the identification of the first COVID-19 case in Japan on 15 January 2020, multiple local COVID-19 clusters were observed. The genome sequences of Japanese isolates were assigned based on the phylogeny of China isolates from late February, but nationwide dissemination seemed to already be present based on a maximum likelihood (ML) phylogeny and dynamic lineage nomenclature for SARS-CoV-2 (10) (Fig. 1).

A SARS-CoV-2 lineage analysis suggested that the B and A clades were initially tracked as the most common variants in Japan, while the B.1.1 clade was recently identified as one of the most active virus lineages in Japan (Fig. 1A). In addition, although at least 4 or 5 main clusters were observed using ML phylogeny (Fig. 1B), this has not always adequately explained the results of field epidemiological studies that were performed by local public health centers using patient information such as nationality, being a Wuhan returnee, and travel history. Although phylogenetic trees are widely used for summarizing genealogies, a naive interpretation of results obtained from phylogenetic trees alone may not provide accurate conclusions. The low rate of SARS-CoV-2 evolution and the sampling bias of genomes can often lead to spurious conclusions (11), suggesting that it does not imply the direction of transmission from one to another.

**FIG 2:**
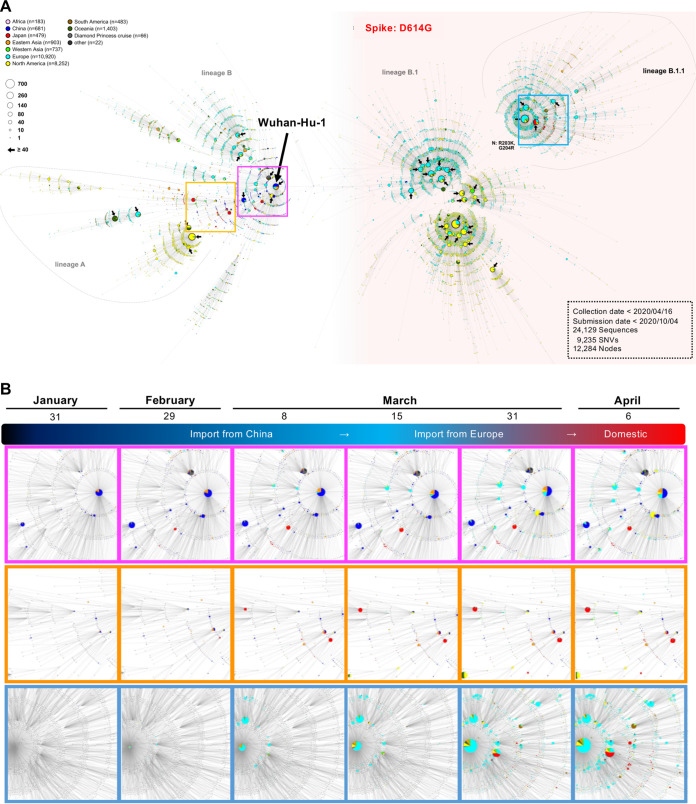
Haplotype network analysis using genome-wide single nucleotide variations of worldwide SARS-CoV-2 isolates. (A) Whole-genome sequences of SARS-CoV-2 isolates in Japan (*n* = 435) were compared to all SARS-CoV-2 genomes available in the GISAID database (*n* = 23,694 [updated on 10 October 2020]). SARS-CoV-2 disseminating from Wuhan City, China, at the end of December 2019 (one of the potential origins of Wuhan-Hu-1) is plotted at the center of the haplotype network. In total, 9,235 SNVs were detected in 24,129 isolates. Isolates carrying the D614G amino acid substitution in the spike protein are highlighted with a pink background. Bold arrows indicate marked large genome clusters consisting of ≥40 entries. The PANGOLIN (A, B, B.1, and B.1.1) is shown beside the cluster nodes. (B) Three plots of time series cumulative COVID-19 cases are highlighted in each enclosed square to visualize the increasing incidence of COVID-19 cases. The timeline movie (mp4 file) is available in the supplemental material (Movie S1).

We selected a total of 24,129 genome sequences, including Japanese isolates, as described above; thus, the large number of candidates included in the phylogenetic analysis (Fig. 1B) obscured details of the routes of transmission of SARS-CoV-2 between patients and among event-specific clusters. Neither a high image resolution nor the image magnification in Fig. 1B worked to identify a marked cluster as regional or event specific. Haplotype network analysis provides a better picture of epidemiology over the short term, such as during the current COVID-19 outbreak, although it does not overcome the issues of biased sampling and limited diversity based on genetic evolution (12). Therefore, we used haplotype network analysis to highlight potential infectious linkages over a very short period (days, weeks, or months) and to demonstrate that regional or event-specific clusters are case number dependent (Fig. 2; see also Movie S1 in the supplemental material).

10.1128/mSphere.00786-20.5 MOVIE S1 Genomic network movie file (mp4 format). Download Movie S1, MOV file, 9.0 MB. Copyright © 2020 Sekizuka et al. 2020 Sekizuka et al. This content is distributed under the terms of the Creative Commons Attribution 4.0 International license.

To decode the genealogies of the whole SARS-CoV-2 genome, we performed a haplotype network analysis describing ancestral relationships between the genomic data sets collected in this study and the Wuhan-Hu-1 genome being the most recent and common potential ancestor (Fig. 2). In total, 9,235 SNVs were detected in 24,129 isolates. Some of the primary clusters identified through January and February in Japan (2 red closed circles in magenta frames in Fig. 2) descended directly from the haplotype that was commonly seen in the Wuhan-Hu-1-related isolates from China. Two other distinct clusters (2 additional red closed circles in orange frames in Fig. 2) were observed after the first introduction of the Chinese isolates. The four clusters were assumed to have originated directly from the primary wave that occurred in China and were related to mass gatherings such as a party in a small area, a snow festival, and a rock concert. These clusters could also be related to nosocomial infections. Mass-gathering-related clusters caused nationwide spread as the people who interacted with COVID-19 patients returned home after attending the events. Most of these clusters were contained by the efforts of local public health centers and through the implementation of active surveillance in Japan until mid-March.

In contrast, the number of COVID-19 cases increased rapidly across Europe and North America during early March (Fig. 2A, right), indicating that a pandemic (mainly Phylogenetic Assignment of Named Global Outbreak Lineages [PANGOLIN] B.1 and B.1.1) was occurring. Concurrently, many sporadic COVID-19 cases were detected in Japan from the end of March to early April. The haplotype network analysis demonstrated that additional large clusters (PANGOLIN B.1.1) (red closed circles and cyan frames in Fig. 2A and B) showed either identical or additional SNVs compared to the original from Europe (cyan closed circles in Fig. 2), although the additional clusters showed eight SNVs from Wuhan-Hu-1 in China (magenta frame), placing it in a distinct phylogenetic lineage (PANGOLIN B in Fig. 1A and Fig. 2A), indicating that the second introduction during early April in Japan did not originate from Wuhan but was closely related to the outbreak in Europe. This observation is supported by the timeline in Movie S1 (mp4 file).

**FIG 3:**
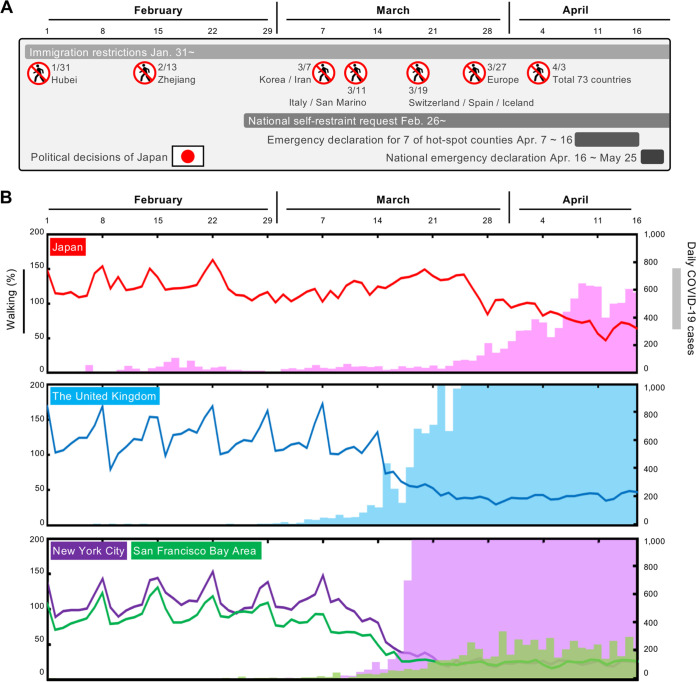
Mobility index (walking) of people and daily COVID-19 cases in Japan, the United Kingdom, New York City, and the San Francisco Bay area. (A) Timeline of political decisions for the COVID-19 quarantine and national actions in Japan (see also Table S4 in the supplemental material). (B) Mobility index of people and daily COVID-19 cases in Japan, the United Kingdom, and the United States (New York City and the San Francisco Bay area). Solid lines indicate the mobility index of people inferred from map application usages given by Apple. The bar plot indicates daily COVID-19 cases. Notably, the data suggest that people in Japan and the San Francisco Bay area collaborated with “stay-at-home” measures from the end of February, which might have reduced the expansion of SARS-CoV-2 infections after late March 2020.

Analysis of whole-genome sequences provides information that is useful in tracking the spread of infection using genome-wide SNVs among clusters. To elucidate which Japanese political decisions (Fig. 3A and Table S4) and activities were effectively involved in the introduction of specific SARS-CoV-2 lineages, we characterized daily COVID-19 case reports and the mobility index of people by tracking iPhones (Fig. 3B) in Japan, the United States, and the United Kingdom both before and after the self-restraint campaign. During the primary wave from China, international airports investigated potential patients with COVID-19 using the keywords “Wuhan, Hubei, Zhejiang, and China” in early February (Fig. 3A). In addition, local public health centers identified patients likely to be infected with COVID-19 and their close contacts within location-specific clusters by conducting active epidemiological surveillance. After the end of February, national self-restraint commenced in Japan, leading to a significant reduction in the spike of mobile activity over weekends. Although the situation in Japan had begun to improve around mid-March, a large number of new patients with COVID-19 were diagnosed, many of whom had unclear infection routes. Tracing the infection routes was difficult because some of these Japanese cases had no recent history of travel to China or any other country outside Japan.

During the national holidays from 20 to 22 March 2020, a part of the population left their homes to visit the cherry blossoms, which possibly increased mobility (Fig. 3B). It was speculated that such a partial increase in activity at that time might have allowed for a resurgence of the remaining lineage from the first introduction that had circulated previously. The SARS-CoV-2 haplotype network analysis, however, suggested that the second wave of COVID-19 cases in late March had a distinctly different origin from the lineage of the first wave. The lineage of the second wave could have been imported by returnees or travelers from Europe, North America, or other countries (13) (see the image in the cyan frame in Fig. 2) but not from the remaining Wuhan lineage. Considering the increasing COVID-19 cases in Europe and the detection of imported cases in Japan, the Japanese government decided to stop immigration from Europe on 27 March 2020. (Fig. 3A). On the other hand, increased mobility in the United States and the United Kingdom continued through early March, possibly leading to a sharp increase in COVID-19 cases from mid-March (Fig. 3B). Intriguingly, the activity in the San Francisco Bay area slowed down earlier than in New York; the additional 3 days taken by New York to implement stay-at-home/shelter-in-place orders compared to San Francisco may explain the mitigated COVID-19 spread in San Francisco.

While Japan did not impose any strict regulations such as a lockdown of any city, national self-restraint was requested of the people on 26 February 2020. This self-restraint is reflected as reduced peak activity during every weekend in Japan (Fig. 3B). Although the self-restraint request is a weak regulation, such long-lasting self-restraint should have been effective in mitigating an increase in COVID-19 cases by mid-March; however, large amounts of immigration and late restrictions on travel from Europe around mid-March are probable reasons for the increase in cases.

The late action on stopping immigration from Europe was the prime reason for the unexpected increase in COVID-19 cases; also, the increase in mobile activity during national holidays during mid-March and the late response in declaring a national emergency may have escalated the outbreak at the local level.

### Conclusions.

This genome surveillance study suggested that there were at least two distinct SARS-CoV-2 introductions into Japan, initially from China and subsequently from other countries, including Europe. Since immigration restriction has been in place since 27 March, we have not identified any additional introduction from abroad thus far. Indeed, the current SARS-CoV-2 isolates in September were classified as the progeny of the Europe-related Japanese isolates (B.1.1 PANGOLIN) that were identified in mid-March 2020. To mitigate the next wave of COVID-19 in Japan, further requests for self-restraint could also help with the containment of local clusters by avoiding further spread across borders, and it is necessary to formulate a more efficient containment strategy using real-time genome surveillance to support epidemiological field investigations in order to highlight potential infection linkages and enable prompt decision-making by health authorities and the government.

## MATERIALS AND METHODS

### Ethical approval and consent to participate.

The study protocol was approved by the National Institute of Infectious Diseases in Japan (approval no. 1091). It was conducted according to the principles of the Declaration of Helsinki, in compliance with the Law Concerning the Prevention of Infections and Medical Care for Patients of Infections of Japan. The ethical committee waived the need for written consent since the study involved the sequencing of viral genomes. Personal data related to clinical information were anonymized, and we did not request written consent from all patients with COVID-19 who were included in the study.

### Clinical specimens and reverse transcription-PCR testing for COVID-19.

Nasopharyngeal specimens were collected from patients, and reverse transcription-quantitative PCR (RT-qPCR) testing for SARS-CoV-2 (14, 15) was performed at local public health institutes in Japan (see Table S1 in the supplemental material). Positive RNA samples were subjected to whole-genome sequencing.

### Whole-genome sequencing of SARS-CoV-2.

Whole-genome sequences of SARS-CoV-2 were obtained by means of the PrimalSeq protocol for enriching the cDNA of the SARS-CoV-2 genome using multiplex RT-PCR, as proposed by The Wellcome Trust ARTIC Network (16). We found two amplicons that regularly showed low to zero coverage due to primer dimerization; therefore, we modified the ARTIC Network’s protocol for SARS-CoV-2 genome sequencing by replacing some of the primers for the multiplex PCR (17). The PCR products in pools 1 and 2 from the same clinical sample were pooled, purified, and subjected to Illumina library construction using a QIAseq FX DNA library kit (Qiagen, Hilden, Germany). The NextSeq 500 platform (Illumina, San Diego, CA) was used for sequencing the indexed libraries. The next-generation sequencing (NGS) reads were mapped to the SARS-CoV-2 Wuhan-Hu-1 reference genome sequence (29.9-kb single-stranded RNA [ssRNA] [GenBank accession no. MN908947]). The specimen-specific SARS-CoV-2 genome sequence was obtained by complete mapping to the reference sequence. The mapped reads of the SARS-CoV-2 sequences were assembled using A5-miseq v.20140604 (18) in order to determine the full genome sequence (Table S2). The SNV sites and marked heterogeneity were extracted by read mapping at a ≥10× depth and from the region spanning nucleotides (nt) 99 to 29796 of the Wuhan-Hu-1 genome sequence. The lineage classification of SARS-CoV-2 was performed using PANGOLIN v2.0 (https://github.com/cov-lineages/pangolin) (10).

10.1128/mSphere.00786-20.2 TABLE S2 Summary of NGS reads and *in silico* data analysis. Download Table S2, XLSX file, 0.05 MB. Copyright © 2020 Sekizuka et al. 2020 Sekizuka et al. This content is distributed under the terms of the Creative Commons Attribution 4.0 International license.

### Comparative genome sequence and SNV analyses.

The nearly full-length complete genome sequence (≥29 kb) of SARS-CoV-2 was retrieved from the GISAID EpiCoV database on 10 October 2020. Simulated short reads of these genome sequences were generated using the SimSeq program (available at https://github.com/jstjohn/SimSeq) with parameters of a 150-mer read length, 200-bp insert size, 0 simulated read errors, and 10,000 paired-end reads, followed by extraction of SNV sites using mapping analysis. The poorly aligned regions at the 5′ and 3′ ends were trimmed; we determined that the core regions were from nt 99 to 29796 against the Wuhan-Hu-1 genome sequences (GISAID accession no. EPI_ISL_402125; GenBank accession no. MN908947.3). Gap-containing sequences in the core region were excluded; sequences of 23,694 isolates in the GISAID database were eventually used in subsequent analyses (isolates were collected by 16 April 2020 and submitted to GISAID by 10 October 2020) (Table S3), and all SNV sites were merged using GATK version 3.8 (19). The genome sequences were aligned with the sequences retrieved from the databases using MAFFT software. This was followed by the extraction of SNV sites. ML phylogenetic analysis with SNVs was performed using FastTree version 2.1.10 (20) with default parameters, followed by visualization using FigTree v1.4.4.

10.1128/mSphere.00786-20.3 TABLE S3 All SARS-CoV-2 genomes available in the GISAID database (*n* = 23,694). Download Table S3, XLSX file, 1.1 MB. Copyright © 2020 Sekizuka et al. 2020 Sekizuka et al. This content is distributed under the terms of the Creative Commons Attribution 4.0 International license.

10.1128/mSphere.00786-20.4 TABLE S4 Japanese immigration restriction programs. Download Table S4, XLSX file, 0.01 MB. Copyright © 2020 Sekizuka et al. 2020 Sekizuka et al. This content is distributed under the terms of the Creative Commons Attribution 4.0 International license.

### Network graph visualization.

An edge list for network data was converted from ML phylogenetic data (i.e., Newick format) using “ape” (21) and the “igraph” (https://igraph.org/) library of the R package; the polytomy was merged into a single node. The pairwise SNV distance matrix was generated using the snp-dists version 0.7 program (https://github.com/tseemann/snp-dists), followed by the extraction of an edge list containing only one base mismatch. Network data were reconstructed using these two edge lists, and duplicate and/or self-edges were removed. The node of network data was positioned by force-directed graph layout with the edge, including the number of SNVs, using D3.js (22), followed by visualization, including metadata information, using Cytoscape version 3.8.0 (23). Over 100 network graph images were captured daily from the initial detection of SARS-CoV-2 (Wuhan-Hu-1, 31 December 2019) to 16 April 2020, the day when Japan declared a national emergency. The movie (mp4 file) is available in the supplemental material (Movie S1).

### Comparison of outdoor activity and numbers of cases in Japan, the United States, and the United Kingdom.

We explored the relationships between outdoor activity, daily COVID-19 cases, and local stay-at-home orders/shelter-in-place orders/self-restraint campaigns in Japan, the United States (New York City and the San Francisco Bay area), and the United Kingdom. The data show the population mobility retrieved from the COVID-19 mobility report provided by Apple (the data are available for a limited time only) (24). The mobile activity was normalized to the absolute value for 13 January 2020 according to the Apple mobility trend report website. In addition, Apple has stated that the absolute value of baseline mobility is not reported on their website as the company does not have the population statistics of every city and country. The values for walking were plotted for the three above-mentioned countries (Japan, the United Kingdom, and the United States). We subsequently plotted the mobility graph using the daily and total COVID-19 cases that were provided by the National Institute of Infectious Diseases (currently the only Japanese data available) (25), coronavirus data in the United States (provided by the New York Times) (26), and coronavirus source data (27).

### Data availability.

The new sequences have been deposited in the Global Initiative on Sharing All Influenza Data (GISAID) database with accession no. EPI_ISL_479792 to EPI_ISL_480227 (see Table S2 in the supplemental material).
